# Hypersensitivity to oxaliplatin: clinical features and risk factors

**DOI:** 10.1186/2050-6511-15-1

**Published:** 2014-01-13

**Authors:** Marie Parel, Florence Ranchon, Audrey Nosbaum, Benoit You, Nicolas Vantard, Vérane Schwiertz, Chloé Gourc, Noémie Gauthier, Marie-Gabrielle Guedat, Sophie He, Eléna Kiouris, Céline Alloux, Thierry Vial, Véronique Trillet-Lenoir, Gilles Freyer, Frédéric Berard, Catherine Rioufol

**Affiliations:** 1 Hospices Civils de Lyon, Clinical Oncology Pharmacy Department, Pierre-Bénite, France; 2 Hospices Civils de Lyon, Clinical Oncology Pharmacy Department, Pierre-Bénite - Université Lyon 1, EMR 3738, Lyon, France; 3 Hospices Civils de Lyon, Allergy and Clinical Immunology Department, Pierre-Bénite, France; 4 Oncologie Médicale, Centre d’Investigation des Thérapeutiques en Oncologie et Hématologie de Lyon (CITOHL), Centre Hospitalier Lyon-Sud, Hospices Civils de Lyon, Lyon - Université Lyon 1, EMR 3738, Lyon, France; 5 Centre Régional de pharmacovigilance de Lyon, France

**Keywords:** Oxaliplatin, Hypersensitivity, Risk factors, Platinum salts, Desensitization

## Abstract

**Background:**

Oxaliplatin-based regimens induce a potential risk of hypersensitivity reaction (HSR), with incidence varying from 10% to 25% and lack of clearly identified risk factors. The present study aimed to assess incidence and risk factors in HSR.

**Methods:**

All patients treated with oxaliplatin in the Medical Oncology Department of the Lyon Sud University Hospital (Hospices Civils de Lyon, France) from October 2004 to January 2011 were enrolled. Incidence and severity of HSR were analyzed retrospectively and the potential clinicopathological covariates were tested on univariate and multivariate analysis.

**Results:**

A total of 1,221 doses of oxaliplatin were administered for 191 patients, 8.9% of whom experienced an HSR. Seventeen HSRs were observed, with 1.6% grade 3 and no grade 4 events. The first reaction appeared after a median of 3 oxaliplatin infusions. Using univariate analysis, HSR was associated with younger age (mean age, 56.2 years; p = 0.04), female gender (p = 0.01) and prior exposure to platinum salts (p = 0.02). No increased risk was associated with mean dose or with presence of atopic background. Multivariate analysis confirmed that women were at higher risk of oxaliplatin HSR than men (p < 0.05). Reintroduction of oxaliplatin was effective in 64.7% of hypersensitive patients using an appropriate premedication strategy. Patients who experienced a grade 3 HSR were not rechallenged.

**Conclusion:**

The risk of developing oxaliplatin HSR should not be underestimated (8.9% of patients). The medical team’s vigilance should be increased with women, younger patients and patients with prior exposure to platinum salts.

## Background

Chemotherapy agents can induce hypersensitivity reaction (HSR), reducing the use of critical drugs in fragile patients for fear of inducing severe reaction and possibly death. However, with improved outcomes in cancer care, longer patient survival and extended treatment courses, patients are exposed to drugs more frequently and for longer periods, increasing the risk of sensitization and of HSR. Prevention and management of acute infusion reactions in oncology remain essential [1], particularly with platinum agents (carboplatin, cisplatin and oxaliplatin) [2]. HSR should be an important concern, due to its potential life-threatening risk and the subsequent treatment withdrawal [3].

Oxaliplatin, a third-generation platinum agent, has been approved for the treatment of metastatic colorectal cancer and for adjuvant treatment in stage-III colon cancer [4]. It is also used worldwide in other malignancies, such as ovarian cancer [5]. As colorectal cancer is the third most common cancer in the world and the second leading cause of cancer death in western countries, its treatment is currently a public health priority and oxaliplatin represents a key chemotherapeutic agent [6].

HSR to oxaliplatin has been less frequently described than to cisplatin and carboplatin. However the increasing use of oxaliplatin in clinical practice shows significant incidence, varying from 10% to 23.8% [3,7-13]. Identifying patients with high risk of oxaliplatin-HSR is a major clinical issue and several studies assessed risk factors, although with heterogeneous results [3,7,9,10,14]. Moreover, Asian populations were mostly investigated [3,7,8,10,12-15], but only some studies evaluated the risk factors using multivariate analysis [3,7,9,12].

The aim of this retrospective cohort study was to investigate the clinical features and the risk factors of oxaliplatin-HSR.

## Methods

### Source of data

All patients who received at least one dose of oxaliplatin in the Medical Oncology Department of the Lyon Sud University Hospital (Hospices Civils de Lyon, France) over a 6-year period from October 2004 to January 2011 were identified using the pharmaceutical software dedicated to antineoplastic preparation. Routine premedication before oxaliplatin infusion included 120 mg of methylprednisolone given intravenously and antiemetic prophylaxis.

The following clinical data were collected from medical files: sex, age, history of allergy, oxaliplatin HSR, type of cancer, previous exposure to platinum agents, and oxaliplatin exposure during the study period (total number of courses, doses and cumulative dose), and treatment line number.

Data from patients who experienced oxaliplatin-associated HSR were compared to that of controls patients who received oxaliplatin without experiencing HSR.

For the purposes of the present study, HSR was defined as any unexpected adverse manifestation including non-allergic drug hypersensitivity and drug allergy reactions like the typical symptoms of IgE-mediated reactions, including cutaneous symptoms such as palmar or facial flushing, respiratory symptoms such as shortness of breath, and cardiovascular symptoms such as any alteration in pulse or blood pressure [16]. Expected side-effects, such as chemotherapy-induced nausea or vomiting and diarrhea, were excluded. Severity was graded according to the Ring and Messmer’s classification [17] and the National Cancer Institute Common Terminology Criteria for Adverse Events (NCI-CTCAE v 4) because of its frequent use by medical oncologists worldwide (Table 1). The outcome data was also recorded for each patient.

**Table 1:** Clinical severity scale of immediate reactions

	**According to ring and messmer **[17]	**According to NCI-CTCAE**
Grade	Clinical signs	
1	Cutaneous-mucous signs	Mild transient reaction with no infusion interruption
2	Cutaneous-mucous signs	Therapy or infusion interruption indicated but responds promptly to symptomatic treatment (antihistamines, corticosteroids, narcotics, IV fluids)
	Cardiovascular signs (tachycardia, hypotension)	
	Respiratory signs	
3	Cardiovascular collapse	Prolonged reaction not rapidly responsive to symptomatic medication and/or brief interruption of infusion.
	Bronchospam	Recurrence of symptoms following initial improvement
		Hospitalization indicated for clinical sequelae
4	Cardiac arrest	Life-threatening consequences with urgent intervention indicated
5	-	Death

NCI-CTCAE: National Cancer Institute Common Terminology Criteria for Adverse Events.

### Statistical analysis

Clinicopathological variables potentially associated with oxaliplatin HSR were subjected to univariate analysis. Statistical analysis was carried out using Wilcoxon’s test to determine whether age or mean dose were associated with hypersensitivity. The other factors (sex, prior platinum exposure and atopic background) were evaluated using Fisher’s exact test.

For multivariate analysis, a logistic regression model was applied. All variables with a p-value ≤ 0.1 on univariate analysis were considered candidates for multiple logistic regression. Two-sided p values exceeding 0.05 were considered statistically significant. Statistical analysis used SAS software, version 8.

Since data were collected retrospectively and that patients’ management was not modified, according to the French law (n°2004-806, 9th august 2004), this study did not need to be approved by a research ethics committee [18]. It was conducted in accordance with the law on data protection (n°2004-801, 6th august 2004).

## Results

A total of 191 patients treated with oxaliplatin were included. Patient characteristics are listed in Table 2. About 59% had colorectal cancer, 70% of whom were treated with a FOLFOX4 regimen (combination of oxaliplatin, 5-fluorouracil and leucovorin). During the 6-year follow-up, 17 patients experienced an HSR to oxaliplatin, representing an incidence of 8.9% of all treated patients (Table 3). A total of 1,221 doses of oxaliplatin were administered, with 1.8% overall frequency of HSR. Patients received median of 4.7 infusions, with a mean 85.3 mg/m^2^ oxaliplatin per infusion. First HSR appeared after median of 3 infusions, and before the 7^th^ course in most patients (80%). Among the 7 patients who developed a first HSR at the 1^st^ or 2^nd^ course, 3 have already been exposed to platinum agents. Sixteen of the 17 patients with HSR were women (94%) and 2 of them were treated for ovarian cancer.

**Table 2:** Patient characteristics

**Patient characteristics (n = 191)**	**Mean (range)**	**n (%)**
Age (years)	62.4 (23–84)	
Sex, male		78 (41)
Atopic diseases		32 (17)
Diagnosis		
Colon		86 (45)
Stomach		10 (5)
Ovary		35 (18)
Pancreas		9 (5)
Peritoneum		8 (4)
Rectum		25 (13)
Other^1^		18 (10)
Prior platinum exposure, yes		45 (24)
Treament regimen		
FOLFOX4 (oxaliplatin, 5-fluorouracil and leucovorin)		101 (53)
FOLFOX4-bevacizumab		10 (5)
FOLFOX4-cetuximab		4 (2)
GEMOX (oxaliplatin and gemcitabine)		47 (25)
Oxaliplatin alone		5 (3)
TOMOX(oxaliplatin and raltitrexed)		12 (6)
Other^2^		12 (6)
Total infusion courses	6.4 (1–18)	
Oxaliplatin dose (mg/m^2^)	85.3 (42–160)	

^1^Includes: appendix (2), gallbladder (4), liver (2), mouth (1), oesophagus (2), endometrium (1) and unknown primary (6).

^2^Includes: 2 ELOGEM (combination of oxaliplatin and gemcitabine), 2 EOX (combination of oxaliplatin, epirubicin and capecitabine), 2 FOLFIRINOX (combination of oxaliplatin, irinotecan, 5-fluorouracil and leucovorin), 1 FOLFOX6 (combination of oxaliplatin, 5-fluorouracil and leucovorin), 2 FOLFOX7 (combination of oxaliplatin, 5-fluorouracil and leucovorin), 1 patient treated with oxaliplatin and epirubicine, 1 oxaliplatin with cetuximab and 1 XELOX (combination of oxaliplatin and capecitabine).

**Table 3:** Hypersensitivity reactions to oxaliplatin

	**Hypersensitivity reactions to oxaliplatin**
Hypersensitivity reactions	17 (8.9%)
Severity according to Ring and Messmer [17]	
Grade 1 events	12 (6.3%)
Grade 2 events	2 (1.0%)
Grade 3 events	3 (1.6%)
Grade 4 events	0
Severity according to to NCI-CTCAE	
Grade 1 events	5 (2.9%)
Grade 2 events	9 (4.7%)
Grade 3 events	3 (1.6%)
Grade 4 events	0
Grade 5 events	0
Cycle number at event	
Median (range)	3 (1–13)

NCI-CTCAE: National Cancer Institute Common Terminology Criteria for Adverse Events.

During the first reaction, the most common effects were flushing (10 patients), urticaria (4 patients) and fever (4 patients). Two patients experienced dyspnea without bronchospasm. Severity of HSRs according to the Ring and Messmer’s classification and the National Cancer Institute Common Terminology Criteria for Adverse Events (NCI-CTCAE v 4) was similar (Table 3). Most reactions were moderate but three patients presented grade 3 HSRs with hypotension, hypothermia and symptomatic larynx spasm (1 patient), acute hypertension, symptomatic larynx spasm and dyspnea (1 patient), and hypotension, bradycardia, hypothermia and fainting (1 patient). There was no grade 4 event (heart failure or respiratory arrest which required urgent intervention) and no death. Nine patients responded promptly to symptomatic treatments (antihistamines, corticosteroids, narcotics or IV fluids) and 3 patients had a more prolonged reaction not rapidly responsive to symptomatic medications and/or brief interruption of infusion.

After their first HSR episode, 13 patients (76%) were rechallenged with a mean 4.8 further cycles of oxaliplatin, associated in most cases with strengthened premedication including antihistamines and higher-dose corticosteroids. Patients who experienced a grade 3 HSR were not rechallenged. Four (30.8%) experienced another HSR at the following course: 1 second reaction in 3 patients and 2 in another patient, which 1 required definitive withdrawn of oxaliplatin. A total 22 HSRs occurred in 17 patients and required definitive withdrawn of oxaliplatin in 2 patients. Clinical signs and severity were generally similar in first and repeat episodes. Three clinicopathological parameters emerged as potential risk factors for oxaliplatin HSR using univariate analysis (Tables 4 and 5). Patients from the HSR group were younger (mean age, 56.2 years) than controls (mean age, 62.6 years; p < 0.05). The rate of oxaliplatin HSR was higher in women than men (p < 0.05), with 14.2% of all women treated with oxaliplatin having reactions compared to only 1.3% of all men. Thirdly, HSR was more frequent in patients previously treated with platinum salts (p < 0.05). Mean oxaliplatin dose and presence of atopic background were not significantly associated with HSR. Using multivariate analysis, sex was the only remaining significant covariate, thereby confirming that women are at higher risk of oxaliplatin than men (p < 0.05).

**Table 4:** Results of univariate analysis (n = 191)

	**Patients (n)**	**HSR (n)**	**HSR (%)**	**p value**^**1**^
Sex				
Female	113	16	14.2	0,0147
Male	78	1	1.3	
Prior exposure to platinum salts				
Yes	45	8	17.8	0,0220
No	146	9	6.2	
Atopic background				
Yes	32	4	12.5	0,4367
No	159	13	8.2	

^1^Fischer exact test.

**Table 5:** Results of univariate analysis (n = 191)

	**Patients with HSR (n = 17)**	**Patients without HSR (n = 174)**	**p value**^**2**^
Mean age (years)	56.2 ±10.5	62.6 ±12	0.0405
Mean oxaliplatin dose (mg/m^2^)	88.5 ±15.2	87.4 ±13.1	0.7473

^2^Wilcoxom’s test.

## Discussion

Hypersensitivity reactions to antineoplastic agents are commonly overlooked. However, for some drugs, particularly L-asparaginase, taxanes and platinum salts, hypersensitivity may lead to the withdrawal of the chemotherapy, thereby reducing the number of therapeutic options. The incidence of oxaliplatin HSR is rising as a consequence of its increased clinical use. Given the limited number of active agents in colorectal malignancy, it is now necessary to understand this kind of reactions and ways of prevent them [19]. The present retrospective study of 191 French patients aimed at bringing new insights to the clinical features of oxaliplatin HSR and its risk factors.

Incidence of oxaliplatin HSR and number of previous cycles appeared to differ from other reports, with lower incidence (8.9%) and earlier onset (before the 6^th^ course), compared to 10–23.8% incidence [3,7-11,13,20] and onset mostly after 6–10 courses [3,10,14,20-22]. As previously described, age and female gender were identified as risk factors [7,9,13]. Interestingly, we showed that 94% of hypersensitive patients were women, finding which was not reported yet. The reasons for this increased risk amongst females are not known. However, it is well established that, for unknown reasons, anaphylaxis is more frequent in women. Kim *et al.* suggest that an association between female gender and younger age may be explained by a possible role of hormonal influences [9]. Another hypothesis could be the important proportion of women (89%) among patients with a previous exposure to platinum agents which is a risk factor of oxaliplatin HSR in univariate analysis in this study. We also investigated the influence of prior exposure to platinum salts. Previous infusions appeared as a risk factor for oxaliplatin HSR. The effect of the platinum-free interval on the incidence and severity of HSR to platinum-containing agents is still a matter of discussion [7]. The impact of concomitant drugs on the risk of HSR is still an issue. In the present study, among 5 patients received oxaliplatin concomitantly with cetuximab, known to induce reaction at the time of administration, 1 patient developed an HSR. Our patients were treated with 14 different regimens which prevent us to assess the involvement of associated treatment due to a lack of power. The efficiency of premedication on HSR is naturally expected. A recent study demonstrated a significant improvement of tolerance with increased doses of dexamethasone and antihistamine [23]. Our patients were systematically treated with 120 mg methylprednisolone before oxaliplatin infusion, leading to a lower incidence of HSR. Several studies assessed the effect of re-exposure to oxaliplatin with or without premedication. In most cases, steroids plus antihistamines and prolongation of oxaliplatin infusion were helpful. A secondary prevention regimen comprising 40 mg famotidine, 20 mg dexamethasone plus 50 mg diphenhydramine was investigated before re-exposure to oxaliplatin in 30 patients with prior HSR: oxaliplatin was well tolerated in 19 of 30 patients (63.3%) for at least 2 cycles [20].

Management of patients with oxaliplatin HSR remains the question of importance in terms of possible therapeutic options. In the present study, 13 of the 17 hypersensitive patients were re-exposed to oxaliplatin; only 4 developed further reactions. Nevertheless, patients who experienced a grade 3 HSR were not rechallenged. Consistently with previous reports, reintroduction of oxaliplatin is generally possible in grade 1/2 hypersensitive patients using an appropriate premedication strategy: i.e. anti-histamine and/or steroids, and reduced infusion flow [24-26]. But uniform approach to prevent oxaliplatin HSR has not been established and reintroduction remains associated with recurrence of HSR, which requires permanent withdrawal of oxaliplatin infusion with obvious harmful consequences for the patient. Efforts strengthening prevention are needed. Consistently with our results, reinforced premedication could be assessed after 3 courses of oxaliplatin (median of our study) for example or for women with prior exposure to platinum salts. Moreover, when oxaliplatin treatment is considered fundamental for the patient after severe HSR, desensitization should be performed. The need to offer first-line therapy has urged the clinical development of rapid desensitization, which allows hypersensitive patients to be re-treated with medications. Such protocols are safe and effective and allow patients to continue with the treatment that initially caused an HSR [27,28].

Finally, another therapeutic option is the switch for carboplatin or cisplatin, in case of gynecological malignancies for example. Successful replacement of carboplatin by cisplatin has been demonstrated but cases of severe reactions have also been reported [29]. The incidence of cross reaction with oxaliplatin is not known and less described. Some authors suggested to perform skin tests to exclude cross-reactivity before substituting one platinum analog for another [30,31]. Skin tests have been reported to predict oxaliplatin allergic HSR [32], but are rarely used in daily practice. Indeed there are time-consuming, uncomfortable for the patient and only available in some drug allergy care centers. Nonetheless, skin tests and the basophile activation test contribute to identifying the mechanism of HSR which remains unclear. Because of HSR usually occurs after multiple infusions, platinum agents are thought to induce a type I response mediated by IgE, followed by the release of histamine and cytokines. Recent studies have suggested the involvement of type II and III reactions [33]. Further analysis of the mechanism may lead to the development of effective therapeutic strategies.

Several limitations of this study should be noted. As this was a retrospective study, hypersensitivity symptoms were not actively pursued. Some medical records were insufficient and the incidence of HSR was probably underestimated. The number of study subjects is small, so data presented should be interpreted with caution.

## Conclusions

Given the frequency and potential severity of oxaliplatin HSR, identification of risk factors could be of therapeutic benefit. In the present analysis, women, younger patients (mean age, 56.2 years) and patients who had experienced a prior exposure to platinum salts presented increased risk. Reintroduction of oxaliplatin is generally possible in grade 1/2 hypersensitive patients using an appropriate premedication strategy but uniform approach to prevent oxaliplatin HSR has not been established and reintroduction remains associated with recurrence of HSR, which requires permanent withdrawal of oxaliplatin infusion. The risk of developing HSR during oxaliplatin treatment should not be underestimated. Patients need to be informed about clinical manifestations, so they can be handled early by physicians and nursing staff. These findings underline that a close monitoring should be systematic during oxaliplatin infusion, especially if risk factors are identified.

## Competing interests

No conflict of interests.

No direct funding was received for this study. The authors were personally salaried by their institutions during the period of writing, although no specific salary was set aside or given for writing the paper.

## Authors’ contributions

MP, FR, AN, BY, TV, VTL, GF, FB, CR have made substantial contributions to conception and design, or acquisition of data, or analysis and interpretation of data; 2) have been involved in drafting the manuscript or revising it critically for important intellectual content; and 3) have given final approval of the version to be published. All authors have been involved in drafting the manuscript or revising it critically for important intellectual content; and 3) have given final approval of the version to be published. All authors have read and approved the manuscript.

## Pre-publication history

The pre-publication history for this paper can be accessed here:

http://www.biomedcentral.com/2050-6511/15/1/prepub
